# Experiencing food insecurity in childhood: influences on eating habits and body weight in young adulthood

**DOI:** 10.1017/S1368980023001854

**Published:** 2023-11

**Authors:** Lise Dubois, Brigitte Bédard, Danick Goulet, Denis Prud’homme, Richard E Tremblay, Michel Boivin

**Affiliations:** 1 School of Epidemiology and Public Health, University of Ottawa, 600 Peter Morand, Ottawa, ON K1G 5Z3, Canada; 2 Université de Moncton, Moncton, NB, Canada; 3 Research Unit on Children’s Psychosocial Maladjustment (GRIP), Université de Montréal, Montréal, QC, Canada; 4 UCD School of Public Health, Physiotherapy and Sports Science, University College Dublin, Dublin, Ireland; 5 École de Psychologie, Université Laval, Québec, QC, Canada

**Keywords:** Food insecurity, Food intake, Body weight, Birth cohort, Longitudinal studies

## Abstract

**Objective:**

To examine how food insecurity in childhood up to adolescence relates to eating habits and weight status in young adulthood.

**Design:**

A longitudinal study design was used to derive trajectories of household food insecurity from age 4·5 to 13 years. Multivariable linear and logistical regression analyses were performed to model associations between being at high risk of food insecurity from age 4·5 to 13 years and both dietary and weight outcomes at age 22 years.

**Setting:**

A birth cohort study conducted in the Province of Quebec, Canada.

**Participants:**

In total, 698 young adults participating in the Québec Longitudinal Study of Child Development.

**Results:**

After adjusting for sex, maternal education and immigrant status, household income and type of family, being at high risk (compared with low risk) of food insecurity in childhood up to adolescence was associated with consuming higher quantities of sugar-sweetened beverages (*ß*_adj_: 0·64; 95 % CI (0·27, 1·00)), non-whole-grain cereal products (*ß*_adj_: 0·32; 95 % CI (0·07, 0·56)) and processed meat (*ß*_adj_: 0·14; 95 % CI (0·02, 0·25)), with skipping breakfast (OR_adj_: 1·97; 95 % CI (1·08, 3·53)), with eating meals prepared out of home (OR_adj_: 3·38; 95 % CI (1·52, 9·02)), with experiencing food insecurity (OR_adj_: 3·03; 95 % CI (1·91, 4·76)) and with being obese (OR_adj_: 2·01; 95 % CI (1·12, 3·64)), once reaching young adulthood.

**Conclusion:**

Growing up in families experiencing food insecurity may negatively influence eating habits and weight status later in life. Our findings reinforce the importance of public health policies and programmes tackling poverty and food insecurity, particularly for families with young children.

Food security is a right for all individuals. The following definition should be kept in mind: ‘Food security exists when all people, at all times, have physical, social and economic access to sufficient, safe and nutritious food which meets their dietary needs and food preferences for an active and healthy life’^(1)^. Even in affluent countries in North America, Europe and Oceania, this right has not been attained for all individuals, and food insecurity remains a prevalent and in many countries a worsening public health problem^(2–4)^. For example, in the USA in 2021, the prevalence of household food insecurity reached 10·2 %. These estimates represent 24·6 million adults and 9·3 million children living in food-insecure households^(5)^. For that same year in Canada, 15·9 % of households were affected by food insecurity to varying degrees^(2)^. For Canadians under 18 years of age, this proportion reached 19·6 %^(2)^. Children and adolescents are particularly vulnerable to the consequences of food insecurity because of their higher nutritional needs for growth and development^(6)^. To some extent, young children may be protected from food scarcity by their parents^(5)^. Nonetheless, all children are affected by the limited food availability within the household^(7)^. They are also likely to feel the stress associated with uncertain access to food for the whole family^(7)^. Among adults, food insecurity tends to affect women more than men^(3)^. Emerging adults who are becoming financially independent are also recognised as a vulnerable group for food insecurity^(8)^.

Food insecurity is associated with poverty and social inequalities^(3)^. Families who are socio-economically disadvantaged and have low purchasing power are susceptible to experiencing transient or prolonged episodes of food insecurity^(9)^. In its most extreme form, food insecurity may translate into missing one or more daily meals, potentially leading to inadequate energy and nutrient intake^(10,11)^. However, even at a marginal or moderate level – that is, experiences ranging from being worried about running out of food to making compromises about the variety, quality or quantity of food consumed – food insecurity is associated with negative diet and health outcomes for individuals, regardless of age^(6,8,12–14)^.

To date, studies exploring food insecurity in relation to dietary or weight outcomes have been conducted using mostly a cross-sectional design^(15,16)^. In many of these studies, food insecurity has been associated with a lower consumption of nutrient-dense foods^(8,16,17)^ and a higher consumption of energy-dense foods of lower nutritional value^(8,18)^. Among children specifically, living in food-insecure households has been related to lower consumption of vegetables^(7,19,20)^, higher consumption of processed foods rich in sugar, fat or Na and poor in dietary fibre^(19–21)^ and higher propensity to skip breakfast^(21,22)^, to eat more snacks^(23)^ and to eat food from fast-food restaurants^(22)^. In relation to weight outcomes, food insecurity has been associated with obesity, mainly among adult women^(24,25)^.

From a life-course perspective, we may hypothesise that early exposure to food insecurity would have a cumulative effect on diet and body weight. Very few longitudinal studies have looked prospectively at the potential long-term influence of food insecurity, when experienced at a young age, on dietary habits or weight status later in life. One recent US population-based longitudinal study (*n* 1568) showed that 14-year-old adolescents (mean age) affected by food insecurity were more susceptible to experiencing food insecurity as young adults^(8)^. This study also reported an association between food insecurity and poorer dietary habits in young adulthood. The association, however, was not influenced by food security status in adolescence^(8)^. Another US longitudinal study (*n* 559) found that food insecurity at age 15 was related to a higher rate of BMI gain in the following 16 years^(26)^. Analyses of sex differences indicated that the association between food insecurity and BMI trajectories over time applied only to women^(26)^. Similarly, a recent longitudinal study of rural American children (*n* 341) reported that a higher gain in BMI from age 9 up to 24 years was predicted by an interaction between household food insufficiency and maternal perceived stress when the child was aged 9 years^(27)^.

Prospective studies for the period between adolescence and adulthood remain limited, and longitudinal studies on food insecurity encompassing the period of early childhood up to adulthood are lacking^(15,20)^. Such studies with a long-term perspective are needed to inform public health policies and to design evidence-based dietary interventions among vulnerable populations affected by limited financial resources, particularly families with young children who are paving the way for future generations. Accordingly, our study aims to examine how food insecurity during childhood up to adolescence relates to dietary and body weight outcomes in young adulthood. We hypothesise that individuals who experienced food insecurity in their early years are, once they reach young adulthood, less likely to make healthy food choices and more likely to skip breakfast and eat evening snacks, to eat meals prepared out of home, to experience food insecurity and to be obese.

## Methods

### Study participants

Our study relies on data from the Québec Longitudinal Study of Child Development (QLSCD)^(28,29)^. This birth-cohort study was designed to investigate how various environmental factors affect children’s cognitive and psychosocial development and wellbeing. At its inception, the QLSCD recruited 2120 children born between October 1997 and July 1998 in the Province of Québec, Canada. To ensure a representation of all public health geographic areas in Québec, participants were randomly selected from the 1997–1998 Master birth register of the province. Children were excluded from the cohort if they were born before 24 or after 42 weeks of gestation, if they suffered from a severe illness or disability at birth, if they were born of a twin pregnancy and if their mother could not communicate in English or French. Participants were first seen when they were aged 5 months, then annually up to age 8, and every 2 years thereafter. After 20 years, 1245 participants (i.e. 58·7 % of the initial cohort) still participated in the 2019 data collection round. Over the years, a vast array of data was collected on multiple aspects of child development (e.g. perinatal information; health and lifestyle; physical, cognitive, and social development and familial and social environments), through interviews, questionnaires, observations and direct measurements. Details about the QLSCD, including data collected at different ages, attrition over the years and main findings, are provided in an earlier publication^(29)^.

### Household food insecurity

When QLSCD participants were aged 4·5, 8, 10, 12 and 13 years, data collection procedures included four questions assessing if families had experienced some degree of food insecurity. These questions were part of a self-administered questionnaire to be answered by the participants’ mothers. Questions covered four dimensions of household food insecurity, namely *food insufficiency* over the last 12 months for a member of the household, and compromises on the variety (*we eat the same thing several days in a row…*), the quantity (*we eat less than we should…*) and the quality (*we can’t provide balanced meals for our children…*) of the food consumed in the household due to financial constraints. More details about these questions are available in the online supplementary material (see online Supplemental Table 1). At a given age (or data collection round), participants with a positive response to any of these four questions were considered to have experienced household food insecurity. These questions were used in earlier QLSCD studies to relate family food insecurity during preschool years to weight status^(30,31)^ and mental health^(32)^ in childhood.

### Dietary and weight outcomes

From March to June 2020, at a mean age (sd) of 22·20 (0·25) years, QLSCD participants were invited to answer an online questionnaire about their usual dietary habits, including the timing of their meals and snacks, the consumption of meals prepared out of home and their frequency of consumption of a list of sixty food items (with quantities, based on choices among three portion sizes). Dietary outcomes derived from this dietary study included skipping breakfast and regularly eating evening snacks, eating meals prepared outside the home (including meals from grocery stores, from all types of restaurants, from cafeterias and from vending machines) at least once a week, eating meals from fast-food restaurants at least once a week, and the relative quantities consumed of sixteen food groups (derived from the sixty food items). For each food item listed in the questionnaire, a relative quantity was determined by multiplying the frequency of consumption reported (converted/d) by a portion size factor (0·5, 1·0 or 1·5, for smaller, average and larger suggested portion sizes, respectively). Each food item was assigned to one of sixteen food groups. Relative quantities of individual food items assigned to a given food group were added up to obtain the relative quantity for that food group.

As part of the online dietary questionnaire, respondents were also asked to report their height and current weight. This information was used to derive two weight outcomes at age 22 years, i.e. the BMI (weight (kg)/height(m)^2^) and the obesity status (BMI ≥ 30·0; yes *v*. no)^(33)^. Because self-reported weight and height are subject to misreporting (i.e. underreporting, for weight and overreporting, for height), the prevalence of obesity might be underestimated^(34)^. To improve the accuracy of reported height and weight compared with measured data, anthropometric data have been corrected using equations derived from published Canadian data (comparing measured and self-reported data) for adults of various age-sex groups^(34)^. For comparison, the present study presents findings from corrected and uncorrected BMI.

Right after the dietary study (in July and August 2020), all QLSCD participants were invited to take part in another study that investigated, among other issues, the question of food insecurity during the first months of the COVID pandemic. Food security status at age 22 years, obtained through this COVID study, was also included among the dietary outcomes of the present study. Three dimensions of the problem were assessed, including *being worried about not having enough food*, *not having enough food* and *having to compromise on the quality or on the variety of the food consumed* because of financial constraints. Participants who gave a positive answer to any of the three questions were considered to have experienced food insecurity during the first months of the pandemic.

### Covariates

Because food insecurity and the outcomes investigated may be experienced differently by men and women^(17)^, the sex of the participants was included as a covariate in the analyses. Food insecurity is also strongly associated with being socio-economically disadvantaged. To assess the independent effect of food insecurity, various socio-economic characteristics of the participants’ family at the beginning of the QLSCD were examined and considered as potential covariates (based on an earlier study with QLSCD children about family food insufficiency and overweight in preschool years^(30)^). Exploratory analyses of bivariate associations between these characteristics and predictor and outcome variables allowed identifying the following covariates to be included in subsequent multivariate analyses: maternal education and immigrant status, household income and type of family.

### Statistical analyses

**Fig. 1:**
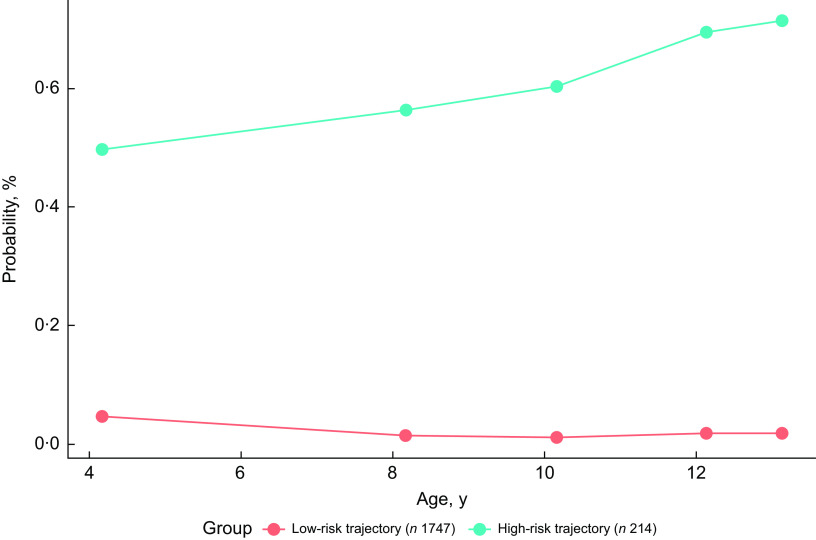
Two group-model trajectories of household food insecurity from age 4·5 to 13 years for QLSCD participants (*n* 1961; probability averages per group). Food insecurity refers to four dimensions that affect access to food: variety (*we eat the same thing several days in a row…*), quantity (*we eat less than we should…*), quality (*we can’t provide balanced meals for our children…*) and food insufficiency (*a member of the family has experienced at least once being hungry …*). QLSCD, Québec Longitudinal Study of Child Development

Supplemental Figure 1 presents a flow chart of QLSCD participants included in different analyses. To explore trajectories of change in food security status from the preschool years up to adolescence, we applied group-based modelling techniques to longitudinal data^(35)^. A total of 1961 QLSCD participants (93 % of the initial cohort) had information about household food security status at least once from age 4·5 to 13 years and were included in these analyses. The group-based trajectory modelling was performed using the ProcTraj procedure^(36)^ in SAS (SAS Institute), version 9.4. Model selection was made in two steps. First, we determined the appropriate number of groups by fitting LOGIT models of 1 to 4 groups with second-order polynomials. We based our decision on the change in the Bayesian Information Criterion between models^(36)^. Second, we determined the shape of each group’s trajectory by setting each group up to a third-order polynomial and removing orders without a significant effect. The final model gave a two-group solution comparing participants who, at a younger age, lived in families at high-risk (11 %) *v*. low-risk (89 %) of food insecurity (Fig. 1). These trajectories of household food insecurity were considered as a predictor in our subsequent analyses.

Our study sample includes 698 QLSCD participants (33 % of the initial cohort) who took part in the dietary study at age 22 years and for whom information about household food insecurity was available at least once from age 4·5 to 13 years. Compared with the rest of the initial cohort, our study sample included a higher proportion of females and participants born in families with a higher socio-economic status (Table 1). No difference was detected between participants and non-participants regarding inclusion in high- or low-risk groups relative to food-insecurity trajectories. Anthropometric data were missing for two participants, and thus analyses regarding weight outcomes included 696 participants. Information on socio-economic covariates was missing for seven participants, yielding a sample size of 691 and 689 for covariate-adjusted models on dietary and anthropometric outcomes, respectively. Exceptionally, analyses of food insecurity at age 22 years included 1174 QLSCD participants (55 % of the initial cohort), for whom we had information on food security status both at age 22 years (i.e. they participated in the COVID study) and during childhood up to adolescence. These 1174 participants were also more likely to be women and individuals from higher socio-economic status families than the rest of the initial cohort (see online Supplemental Table 2).

**Table 1:** Characteristics of the children participating in the QLSCD birth cohort: comparison between participants included in the analyses* and the rest of the initial cohort

Characteristics	QLSCD participants (*n* 2120)		Included in the analyses (*n* 698)		Not included in the analyses (*n* 1422)		*P*-value
	%	*n*	%	*n*	%	*n*	
Sex							
Male	50·9	1080	34·8	243	58·9	837	<0·001
Female	49·1	1040	65·2	455	41·1	585	
Maternal education†							<0·001
Secondary school diploma or less	44·3	940	37·6	262	47·7	678	
Post-secondary and university diploma	55·5	1177	62·4	435	52·3	742	
Data missing	0·1	3	−	1	−	2	
Annual household income (CAD)†							
< 30 000 $	29·3	622	22·7	157	33·5	465	<0·001
≥ 30 000 $	68·9	1460	77·3	536	66·5	924	
Data missing	1·8	38	−	5	−	33	
Immigrant mother†							
Yes	11·9	253	9·0	63	13·4	190	0·005
No	88·0	1865	91·0	634	86·6	1231	
Data missing	0·1	2	−	1	−	1	
Family type†							
Two-parent	80·5	1706	84·2	586	79·1	1120	0·008
Stepfamily	11·1	235	10·1	70	11·7	165	
Single parent	8·1	171	5·7	40	9·3	131	
Data missing	0·4	8	−	2	−	6	
Food-insecurity trajectories (4·5 to 13 years)							
High risk	10·1	214	9·9	69	11·5	145	0·313
Low risk	82·4	1747	90·1	629	88·5	1118	
Data missing	7·5	159	−	−	−	159	

QLSCD, Québec Longitudinal Study of Child Development; CAD, Canadian dollar.

* Includes participants in the dietary study at age 22 years for whom we have information on food insecurity from age 4·5 to 13 years.

† Information collected when children were aged 5 months.

Descriptive statistics include proportions, mean (sd) and median (IQR). *χ* ^2^ tests, one-way ANOVA and Wilcoxon rank sum tests were used to compare proportions, means of normally distributed variables and medians of non-normally distributed variables, respectively. Regression analyses were used to model associations between being at high risk of household food insecurity from age 4·5 to 13 years (compared with being at low-risk, i.e. the reference category) and both dietary and weight outcomes at age 22 years (linear regression for continuous outcomes, i.e. relative quantity of food and BMI; logistical regression for categorical outcomes, i.e. breakfast skipping, evening snacking, eating meals prepared out of home, experiencing food insecurity and obesity). Beta coefficients or OR with 95 % CI and p-values are presented for non-adjusted, sex-adjusted and fully adjusted models (i.e. additional adjustments for maternal education and immigrant status, household income and type of family at the beginning of the QLSCD study). Because we had a few missing values for covariates (≤ 0·015 %), complete case analyses were performed. In linear and logistical regression related to weight outcomes, stratified analyses according to sex were explored to assess a potential moderating effect of sex, as suggested in the scientific literature^(24,25)^. The statistical significance cut-off was set at 0·05. Except for group-based trajectory modelling, all statistical analyses were performed using RStudio^(37)^ with R Statistical Software^(38)^ version 4.2.0.

## Results

As indicated in Table 2, male participants were slightly more than one-third of our sample. This proportion is similar across trajectories of food insecurity from childhood to adolescence. Differences were noted, however, for several other characteristics of the participants. Compared with participants at low-risk of food insecurity, those with a high-risk trajectory were more likely to live alone (20 % *v*. 6 %; *P* < 0·05), to have the lowest level of education (41 % *v*. 17 %; *P* < 0·05), to have a higher BMI (mean (sd): 27·7 (6·5) *v*. 25·6 (5·9); *P* = 0·004) and to be obese (32 % *v*. 16 %, *P* < 0·05*)* at age 22 years. Participants in the high-risk group for food insecurity in childhood up to adolescence were also more likely, as young adults, to have experienced food insecurity during the COVID pandemic (35 % *v*. 12 %; *P* < 0·001), to skip breakfast (30 % *v*. 19 %; *P* = 0·035), to consume evening snacks (68 % *v*. 55 %; *P* < 0·045), to eat at least once a week meals prepared outside the home (91 % *v*. 74 %; *P* = 0·002), namely from fast-food restaurants (64 % *v*. 50 %; *P* = 0·047), and to consume higher relative quantities of sugar-sweetened beverages (median (IQR): 0·86 (2·09) *v*. 0·46 (1·04); *P* < 0·001) and processed meat (0·46 (0·67) *v*. 0·28 (0·50); *P* = 0·002), and lower relative quantities of wholegrain cereal products (0·43 (1·28) *v*. 0·71 (1·07); *P* = 0·030), legumes, nuts and seeds (0·29 (0·57) *v*. 0·56 (0·93); *P* = 0·029) and alcohol (0·00 (0·28) *v*. 0·14 (0·49); *P* = 0·001).

**Table 2:** Comparisons between trajectories of household food insecurity from age 4·5 to 13 years for various characteristics of the participants at age 22 years*

Characteristics (22 years)			Food-insecurity trajectories (4·5–13 years)				*P* value||
	All		Low risk		High risk		
	%		%		%		
Sex – M	34·8		34·7		36·2		0·899
Living situation							<0·001
Alone	7·8		6·4		20·3		
With parents	48·0		49·1		37·7		
With partner, with or without children	25·0		25·2		23·2		
Other situations (incl. combinations)	19·3		19·3		18·8		
Highest level of education							<0·001
Secondary school or less	19·0		16·6		40·6		
College studies	25·7		25·2		30·4		
University studies	55·3		58·2		29·0		
Weight status†							0·001
No excess weight (BMI < 25)	56·0		57·7		40·6		
Overweight (BMI 25·0–29·9)	26·9		26·8		27·5		
Obesity (BMI ≥ 30·0)	17·1		15·5		31·9		
Experiencing food insecurity‡	14·6		12·2		35·0		<0·001
Breakfast skipping on a regular basis	20·1		18·9		30·4		0·035
Snacking in the evening on a regular basis	56·0		54·7		68·1		0·045
Meals prepared out of home: ≥ once/week	75·6		73·9		91·3		0·002
Meals from fast-food restaurants: ≥ once/week	51·7		50·4		63·8		0·047
	Mean	sd	Mean	sd	Mean	sd	
BMI†	25·8	6·0	25·6	5·9	27·7	6·5	0·004
	Median	IQR	Median	IQR	Median	IQR	
Sugar-sweetened beverages§	0·50	1·08	0·46	1·04	0·86	2·09	<0·001
Sweet snacks and desserts§	0·64	0·85	0·64	0·85	0·43	0·87	0·156
Fatty/salty snacks§	0·43	0·72	0·43	0·71	0·57	0·86	0·208
Juices/smoothies unsweetened§	0·43	1·00	0·43	1·00	0·44	0·93	0·153
Fruit§	1·00	1·57	1·00	1·57	0·79	1·21	0·093
Vegetables§	1·78	1·65	1·85	1·71	1·50	1·44	0·067
Non-wholegrain cereal products§	1·10	1·11	1·07	1·06	1·28	1·23	0·098
Wholegrain cereal products§	0·69	1·07	0·71	1·07	0·43	1·28	0·030
Processed meat (incl. pizza and fried chicken/fish/shellfish)§	0·28	0·51	0·28	0·50	0·46	0·67	0·002
Red meat§	0·29	0·43	0·29	0·43	0·29	0·36	0·688
Poultry/fish/shellfish (excl. fried) and eggs§	0·71	0·65	0·71	0·70	0·65	0·61	0·326
Legumes, nuts and seeds§	0·50	0·93	0·56	0·93	0·29	0·57	0·029
Milk and plant-based drinks, unsweetened§	0·29	0·71	0·29	0·71	0·29	0·71	0·901
Cheese§	0·43	0·64	0·43	0·64	0·28	0·72	0·075
Yogurt§	0·07	0·36	0·07	0·36	0·07	0·29	0·873
Alcohol§	0·14	0·43	0·14	0·49	0·00	0·28	0·001

* *n* 698 unless otherwise stated (high-risk trajectory, *n* 69); mean age (sd): 22·2 (0·25).

† *n* 696 (high-risk trajectory, *n* 69); BMI was calculated as kg/m^2^ based on self-reported height and weight corrected^(34)^.

‡ *n* 1174 participants in a special study about COVID conducted in July and August 2020 (high-risk trajectory, *n* 120).

§ Relative quantity consumed/d.

|| Based on the following tests: *χ* ^2^ for proportions, one-way ANOVA for means and Wilcoxon rank sum test for medians.

Results of regression analyses for dietary outcomes are presented in Table 3. In multivariable models adjusted for sex, maternal education and immigrant status, household income and type of family, being on a high-risk trajectory of household food insecurity from childhood to adolescence, was associated with consuming higher quantities of sugar-sweetened beverages (*ß*_adj_: 0·64; 95 % CI (0·27, 1·00)), non-whole-grain cereal products (*ß*_adj_: 0·32; 95 % CI (0·07, 0·56)) and processed meat (*ß*_adj_: 0·14; 95 % CI (0·02, 0·25)), with skipping breakfast (OR_adj_: 1·97; 95 % CI (1·08, 3·53)), with eating meals prepared out of home at least once a week (OR_adj_: 3·38; 95 % CI (1·52, 9·02)) and with reporting continued food insecurity (OR_adj_: 3·03; 95 % CI (1·91, 4·76)) in young adulthood.

**Table 3:** Associations between being at high risk* of household food insecurity from age 4·5 to 13 years and dietary outcomes at age 22 years

Outcome at age 22 years†	Unadjusted models			Sex-adjusted models			Fully adjusted models§		
Relative quantity consumed/d	*ß*	95 % CI	*P* value	*ß*	95 % CI	*P* value	*ß*	95 % CI	*P* value
Sugar-sweetened beverages	0·82	0·46, 1·20	<0·001	0·81	0·46, 1·20	<0·001	0·64	0·27, 1·00	<0·001
Sweet snacks and desserts	−0·06	−0·26, 0·13	0·528	−0·06	−0·26, 0·13	0·513	−0·06	−0·27, 0·14	0·529
Fatty/salty snacks	0·14	−0·03, 0·31	0·102	0·14	−0·03, 0·31	0·105	0·14	−0·04, 0·32	0·120
Juices/smoothies unsweetened	0·19	−0·03, 0·41	0·098	0·19	−0·04, 0·41	0·101	0·12	−0·11, 0·36	0·305
Fruit	−0·33	−0·63, −0·02	0·037	−0·32	−0·63, −0·02	0·039	−0·29	−0·61, 0·04	0·082
Vegetables	−0·29	−0·68, 0·10	0·149	−0·29	−0·68, 0·11	0·153	−0·24	−0·65, 0·17	0·257
Non-wholegrain cereal products	0·32	0·09, 0·55	0·007	0·32	0·09, 0·55	0·007	0·32	0·07, 0·56	0·011
Wholegrain cereal products	−0·14	−0·34, 0·06	0·160	−0·14	−0·34, 0·05	0·152	−0·12	−0·33, 0·09	0·265
Processed meat (incl. pizza & fried chicken/fish/shellfish)	0·19	0·07, 0·31	0·001	0·19	0·08, 029	<0·001	0·14	0·02, 0·25	0·020
Red meat	0·04	−0·05, 0·13	0·384	0·04	−0·05, 0·12	0·404	0·00	−0·09, 0·09	>0·900
Poultry/fish/shellfish (excl. fried) & eggs	−0·15	−0·35, 0·05	0·144	−0·16	−0·35, 0·04	0·122	−0·12	−0·30, 0·06	0·185
Legumes, nuts and seeds	−0·18	−0·37, 0·00	0·055	−0·18	−0·37, 0·00	0·056	−0·14	−0·34, 0·06	0·166
Milk and plant-based drinks, unsweetened	0·14	−0·07, 0·34	0·194	0·13	−0·07, 0·34	0·200	0·13	−0·09, 0·35	0·241
Cheese	0·04	−0·12, 0·19	0·632	0·04	−0·12, 0·19	0·642	0·08	−0·09, 0·24	0·355
Yogurt	0·03	−0·06, 0·11	0·497	0·03	−0·06, 0·11	0·501	0·02	−0·07, 0·11	0·618
Alcohol	−0·16	−0·29, −0·03	0·016	−0·16	−0·29, −0·03	0·014	−0·11	−0·24, 0·03	0·123
	OR	95 % CI	*P* value	OR	95 % CI	*P* value	OR	95 % CI	*P* value
Breakfast skipping on a regular basis	1·88	1·06, 3·21	0·025	1·87	1·06, 3·22	0·026	1·97	1·08, 3·53	0·024
Snacking in the evening on a regular basis	1·77	1·05, 3·06	0·035	1·77	1·05, 3·05	0·035	1·68	0·97, 3·00	0·072
Meals prepared out of home: ≥ once/week	3·70	1·70, 9·72	0·003	3·70	1·70, 9·72	0·003	3·38	1·52, 9·02	0·006
Meals from fast-food restaurants: ≥ once/week	1·73	1·04, 2·94	0·037	1·73	1·04, 2·93	0·038	1·50	0·87, 2·63	0·150
Experiencing food insecurity‡	3·86	2·53, 5·84	<0·001	3·88	2·54, 5·88	<0·001	3·03	1·91, 4·76	<0·001

* Based on two group-model trajectories (reference category: low-risk of household food insecurity).

† *n* 698 unless otherwise stated (high-risk trajectory, *n* 69). For continuous variables, analyses based on linear regressions; for categorical variables, analyses based on logistical regressions.

‡ *n* 1174 participants in a special study about COVID conducted in July and August 2020 (high-risk trajectory, *n* 120).

§ Analyses adjusted for sex, maternal education and immigrant status, household income and type of family when QLSCD participants were children (*n* 691).

Results of regression analyses for weight outcomes using corrected anthropometric data^(34)^ are presented in Table 4. The positive association between a high-risk trajectory of food insecurity from 4·5 to 13 years and BMI at 22 years was detected in non-adjusted and sex-adjusted models but disappeared when adjusting for other covariates (*ß*_adj_: 1·30; 95 % CI (–0·23, 2·90)). However, the positive association appeared to be maintained for extreme (upper) values of BMI. Fully adjusted models indicated that participants who followed a high-risk trajectory of food insecurity across childhood and adolescence were twice as likely to be obese (OR_adj_: 2·01; 95 % CI (1·12, 3·64)) in young adulthood, compared with those at low risk of food insecurity. Stratified analysis according to sex (Table 4) indicates that the positive association detected between food insecurity and obesity is limited to female participants (OR_adj_: 2·93; 95 % CI (1·40, 5·98) *v*. in male participants, OR_adj_: 0·96; 95 % CI (0·28, 2·78)). Using uncorrected BMI gave similar results (see online Supplemental Table 3), although in analyses combining men and women, the positive association for obesity did not reach statistical significance when controlling for socio-economic characteristics.

**Table 4:** Associations between being at high risk* of household food insecurity from age 4·5 to 13 years and weight status at age 22 years

Outcome at age 22 years	Sex	Unadjusted models			Sex-adjusted models			Fully adjusted models||		
		*ß*	95 % CI	*P* value	*ß*	95 % CI	*P* value	*ß*	95 % CI	*P* value
BMI†	All‡	2·20	0·70, 3·70	0·004	2·20	0·70, 3·70	0·004	1·30	−0·23, 2·90	0·096
	Male§	0·71	−1·74, 3·16	0·569				0·58	−2·03, 3·19	0·662
	Female§	3·03	1·15, 4·91	0·002				1·82	−0·14, 3·77	0·068
		OR	95 % CI	*P*-value	OR	95 % CI	*P*-value	OR	95 % CI	*P*-value
Obesity (BMI ≥ 30)	All‡	2·56	1·47, 4·44	<0·001	2·55	1·47, 4·43	<0·001	2·01	1·12, 3·64	0·020
	Male§	1·04	0·33, 2·75	0·938				0·96	0·28, 2·78	0·944
	Female§	4·06	2·05, 7·89	<0·001				2·93	1·40, 5·98	0·004

* Based on two group-model trajectories (reference category: low-risk of household food insecurity).

† BMI was calculated as kg/m^2^ based on self-reported height and weight corrected^(34)^.

‡ *n* 696 unless otherwise stated (high-risk trajectory, *n* 69). Analyses are based on linear regressions for BMI and on logistical regressions for obesity.

§ Male *n* 242; female *n* 454.

|| Analyses adjusted for sex, maternal education and immigrant status, household income and type of family when QLSCD participants were children (*n* 689).

## Discussion

The present study identified two trajectories of food insecurity from age 4·5 to 13 years among the participants of the QLSCD birth cohort. These trajectories defined groups at high *v.* low risk of food insecurity in the early years. As predicted, participants at increased risk of food insecurity in childhood up to adolescence later reported less healthy dietary habits in young adulthood, compared with participants in the low-risk group trajectory. Specifically, they were more likely to skip breakfast and to eat meals prepared out of the home. They also had higher intakes of sugar-sweetened beverages, refined cereal products and processed meat, all of which refer, for the most part, to processed foods that are energy dense, rich in sugar, fat or Na and poor in dietary fibre. Furthermore, these participants in the high-risk group trajectory appeared to be more vulnerable to experiencing food insecurity in young adulthood, at least in the context of the COVID pandemic. Obesity at age 22 years was also more prevalent among those at high risk of food insecurity in childhood up to adolescence, compared with others. However, this last association applied only to women. All these associations were independent of socio-economic characteristics such as maternal education and immigrant status, household income and type of family in early childhood.

Another longitudinal study reported that adolescents living in families affected by food insecurity were more susceptible to experiencing food insecurity once reaching young adulthood^(8)^. Based on food-insecurity trajectories that go back to early childhood, our observations support and extend these findings. Altogether, they suggest that experiencing food insecurity is not only a consequence of a myriad of factors but also becomes, by itself, a factor that may contribute to perpetuating the problem later in life. Although the association was independent of other socio-economic characteristics of the participants at a young age, we noted that young adults in the high-risk group for food insecurity in childhood up to adolescence were more likely to live alone and to have a lower educational attainment than others. Such characteristics may indicate precarious living conditions that might exacerbate the risk of food insecurity in young adulthood.

The results relative to other dietary outcomes confirm previous findings, for the most part from cross-sectional studies, supporting, to some extent, an inverse relationship between food insecurity and diet quality^(15–18)^. We did not detect an independent association between food insecurity and a lower consumption of nutrient-dense foods (e.g. vegetables and fruit, wholegrain cereal products, poultry, fish/shellfish and eggs, dairy products and legumes, nuts and seeds). However, young adults who were at high-risk of food insecurity in their early years had, compared with others, a higher consumption of energy-dense foods of lower nutritional value (e.g. sugar-sweetened beverages, refined cereal products and deli meat, pizza and fried food). Processed foods rich in fat, Na and sugar are known to be largely available and less expensive, on a per-calorie basis^(39)^. Because food prices are among important determinants of dietary choices, these energy-dense foods may become an attractive choice for meeting energy needs of families with limited financial resources^(39,40)^. Highly palatable foods are also suspected to have addictive properties^(41)^. It has been suggested that their consumption may help relieve, at least temporarily, the psychological stress associated with food insecurity^(42)^. Children growing up in food-insecure households where these food choices may have been more readily available are likely to develop a preference for palatable foods and to maintain their consumption over time. This could explain, to some extent, our finding of an independent association between trajectories of food insecurity in childhood up to adolescence and consumption of energy-dense food later in young adulthood.

We found that young adults with a high-risk trajectory of food insecurity were more likely to adopt eating habits such as skipping breakfast and consuming food prepared outside of the home. In other studies, a lower frequency of breakfast consumption has already been noted among children, adolescents and young adults living in food-insecure households compared with those living in food-secure households^(8,21,22)^. Similarly, a recent cross-sectional study conducted among Canadian young adults reported that women from food-insecure households had a lower proportion of meals prepared at home over a 7-d period compared with other women^(43)^. It was suggested that the familial context related to food insecurity, namely potential time constraints related to precarious employment, may contribute, in some instances, to relying on ready-to-eat food instead of cooking meals at home^(43)^. In our study, evening snacking and eating meals from fast-food restaurants were also associated with food-insecurity trajectories in unadjusted and sex-adjusted models. However, these associations disappeared when adjusting for socio-economic characteristics, suggesting that the relationship was dependent on the socio-economic conditions that often co-occur with food insecurity.

Longitudinal studies investigating long-term associations between food insecurity and similar dietary outcomes are limited. As part of a recent population-based, longitudinal study conducted in the USA, cross-sectional analyses adjusted for various socio-economic characteristics indicated positive associations between food insecurity and both skipping breakfast (at least twice a week) and frequently eating fast food (at least 3 times a week) among young adults^(8)^. Participants experiencing food insecurity in young adulthood also ate meals prepared at home less frequently, had lower intakes of nutrient-dense foods (e.g. vegetables, fruit and wholegrain cereal products) and had higher intakes of sugar-sweetened beverages, added sugar and saturated fat, compared with others^(8)^. However, when considering food-security status in adolescence in their models, the authors concluded that they could not find evidence for the impact of food-security status earlier in life on various high-risk health behaviours (including diet-related outcomes) in emerging adulthood^(8)^. Our study used a different approach to look at longitudinal association, which makes comparisons difficult. We also used a larger definition (i.e. including moderate dimensions of food insecurity) and a different time frame for the assessment of food-security status in early years (i.e. a trajectory based on multiple points from childhood to adolescence compared with a single point in adolescence). More long-term longitudinal studies are warranted to better understand how food insecurity experienced early in life influences various aspects of dietary habits later in life.

Our findings related to weight outcomes add to the evidence that food insecurity is associated with obesity mainly among women^(24,25)^. Although obesity is a multifactorial chronic health problem, one potential mechanism for weight gain in the context of food insecurity comes down to a low-quality diet in a stressful environment, which translates into a high consumption of hyperpalatable foods leading to overconsumption of energy and increased visceral adiposity^(44,45)^. In food-insecure households, fluctuations between periods of food scarcity and food availability may also favour the development of eating behaviours such as overeating, which contribute to increased energy intakes^(46)^. Interestingly, in an earlier QLSCD study conducted during the preschool years, we had reported an association between family food insufficiency and a higher risk of overweight and obesity^(30)^. At the time, there was no difference detected between boys and girls in multivariate analyses^(30)^. However, it has been suggested that these sex differences begin to be noticeable later in childhood^(25)^. More research is needed to better understand why the association between food insecurity and weight status would differ across sex. Nevertheless, men and women might perceive and experience differently the stressful conditions associated with food insecurity, which may contribute to gender-based differences^(21)^.

To our knowledge, this is the first study to explore the long-term association between food insecurity and dietary habits, spanning from early childhood up to adulthood. Food insecurity has been assessed at multiple ages in the QLSCD, which allowed us to determine trajectories over time. However, our trajectories did not allow accounting for more nuanced degrees of food insecurity, nor for transient *v*. persistent food-security problems. Also, our results may not be generalisable to the Quebec (or Canadian) population as a whole, as our study sample underrepresented people in socio-economically disadvantaged groups, as well as men. Stratified complementary analyses according to sex did allow, however, to explore sex differences. It also must be recognised that self-reporting methods for dietary assessment and anthropometric measurements are prone to misreporting errors^(34,47)^. For example, BMI tends to be underestimated when using self-reported height and weight^(34)^. We applied validated correction equations to our self-reported data to better approximate BMI based on measured values^(34)^. In this case, results obtained from analyses using corrected and uncorrected anthropometric data led to similar findings.

Although our analyses included adjustments for important characteristics of the participants’ familial contexts, other perinatal factors (e.g. birth weight) and familial factors (e.g. maternal distress when the child was younger, parent’s weight status) were not accounted for, which may contribute to residual confounding. We chose to limit the number of covariates (i.e. sex and socio-economic characteristics) as a compromise to preserve the statistical power of the study. Still, our analyses would deserve to be replicated with a larger sample size, as the numbers for our high-risk group for food insecurity remain relatively small.

In conclusion, our study suggests that growing up in families experiencing food insecurity may have long-lasting effects on eating habits and weight status. Additional research is needed to deepen our understanding of the cumulative effects and underlying mechanisms by which food insecurity contributes to dietary and weight outcomes in the long term. Meanwhile, our findings reinforce the importance of protecting young families affected by food insecurity, with interventions tailored to their needs, as part of strategies aiming to promote healthy eating and healthy weight. Most of all, comprehensive policies and programmes addressing economic issues related to poverty are crucial to ensure food security for all.

## Supplementary material

For supplementary material accompanying this paper visit https://doi.org/10.1017/S1368980023001854. click here to view supplementary material
