# Supplementary material for: Experiencing food insecurity in childhood: influences on eating habits and body weight in young adulthood
Source: Public Health Nutr. 2023 Sep 4;26(11):2396–406. doi: 10.1017/S1368980023001854 (PMC10641605; doi:10.1017/S1368980023001854)
Supplement: Supplementary file 1 [file S1368980023001854sup001.docx]

**Supplemental Table 1** Questions* used in QLSCD to determine household food security/insecurity status of the participants from age 4.5 to 13 years

| Dimension  of food insecurity | Question | Possible responses |
| --- | --- | --- |
| Food insufficiency | **In the past 12 months**, has a member of your family ever experienced being hungry because the family had run out of food or money to buy food? | **Yes**  Yes, regularly, once a month  Yes, more than once a month  Yes, on certain months only  Yes, occasionally, but not regularly  **No**  No |
| Compromise on variety of food consumed | We eat the same thing several days in a row because we have only a few different kinds of food on hand, and we don’t have enough money to buy more. | **Yes**  Often true  Sometimes true  **No**  Never true |
| Compromise on quantity of food consumed | We eat less than we should because we don’t have enough money for food. | **Yes**  Often true  Sometimes true  **No**  Never true |
| Compromise on quality of food consumed | We can’t provide balanced meals for our children because we can’t afford it financially. | **Yes**  Often true  Sometimes true  **No**  Never true |

QLSCD, Québec Longitudinal Study of Child Development

*These questions were part of a self-administered questionnaire to be answered by the participants’ mothers. At a given age (or data-collection round), participants with a positive response to any of these four questions were considered as having experienced household food insecurity.

Quebec Longitudinal Study of Child Development (QLSCD) participants

5 months: *n* 2120

4.5 y: *n* 1944 (91.7%)

8 y: *n* 1451 (68.4%)

10 y: *n* 1334 (62.9%)

12 y: *n* 1396 (65.9%)

13 y: *n* 1290 (60.8%)

Information on household food insecurity − 4.5 to 13 y: *n* 1961 (92.5%)

20 y: *n* 1245 (58.7%)

COVID study

(July-August 2020)

22 y: *n* 1182 (55.8%)

Dietary study

(March to June 2020)

22 y: *n* 700 (33.0%)

Missing data

- trajectories of household food insecurity – 4.5 to 13 y: *n* 2
- weight status – 22 y: *n* 2

Missing data

- trajectories of household food insecurity – 4.5 to 13 y: *n* 8

Final sample for:

- dietary outcomes at 22 y: *n* 698 (32.9%)
- weight outcomes at 22 y: *n* 696 (32.8%)

Final sample for:

- food insecurity outcome at 22 y: *n* 1174 (55.4%)

**Supplemental Figure 1** Flow chart of QLSCD participants included in different analyses

**Supplemental Table 2** Characteristics of the children participating in the QLSCD birth cohort: comparison between participants in the COVID Study at age 22 years about whom we have information on food security status in childhood and the rest of the initial cohort

| Characteristics | QLSCD participants  (*n* 2120) | | Included in the analyses of food insecurity at 22 y (*n* 1174) | | Not included in the analyses of food insecurity at 22 y (*n* 946) | | *P*-value |
| --- | --- | --- | --- | --- | --- | --- | --- |
|  | **%** | *n* | **%** | *n* | **%** | *n* |  |
| Sex |  |  |  |  |  |  |  |
| Male | 50.9 | 1080 | 40.0 | 470 | 64.5 | 610 | <0.001 |
| Female | 49.1 | 1040 | 60.0 | 704 | 35.5 | 336 |  |
|  |  |  |  |  |  |  |  |
| Maternal education^*^ |  |  |  |  |  |  | <0.001 |
| Secondary-school diploma or less | 44.3 | 940 | 39.3 | 461 | 50.7 | 479 |  |
| Post-secondary and university diploma | 55.5 | 1177 | 60.7 | 712 | 49.3 | 465 |  |
| Data missing | 0.1 | 3 | − | 1 | − | 2 |  |
|  |  |  |  |  |  |  |  |
| Annual household income (CAD) ^*^ |  |  |  |  |  |  |  |
| < 30 000 $ | 29.3 | 622 | 25.3 | 294 | 35.7 | 328 | <0.001 |
| ≥ 30 000 $ | 68.9 | 1460 | 74.7 | 870 | 64.3 | 590 |  |
| Data missing | 1.8 | 38 | − | 10 | − | 28 |  |
|  |  |  |  |  |  |  |  |
| Immigrant mother^*^ |  |  |  |  |  |  |  |
| Yes | 11.9 | 253 | 9.2 | 108 | 15.3 | 145 | <0.001 |
| No | 88.0 | 1865 | 90.8 | 1065 | 84.7 | 800 |  |
| Data missing | 0.1 | 2 | − | 1 | − | 1 |  |
|  |  |  |  |  |  |  |  |
| Family type^*^ |  |  |  |  |  |  |  |
| Two-parent | 80.5 | 1706 | 83.2 | 974 | 77.8 | 732 | <0.001 |
| Stepfamily | 11.1 | 235 | 10.8 | 127 | 11.5 | 108 |  |
| Single-parent | 8.1 | 171 | 6.0 | 70 | 10.7 | 101 |  |
| Data missing | 0.4 | 8 | − | 3 | − | 5 |  |
|  |  |  |  |  |  |  |  |
| Food-insecurity trajectories (4.5 to 13 y) |  |  |  |  |  |  |  |
| High-risk | 10.1 | 214 | 10.2 | 120 | 11.9 | 94 | 0.055 |
| Low-risk | 82.4 | 1747 | 89.8 | 1054 | 88.1 | 693 |  |
| Data missing | 7.5 | 159 | − | − | − | 159 |  |
|  |  |  |  |  |  |  |  |

QLSCD, Québec Longitudinal Study of Child Development; CAD, Canadian dollar.

^*^ Information collected when children were aged 5 months.

**Supplemental Table 3** Associations between being at high-risk^*^ of household food insecurity from age 4.5 to 13 years and weight status at age 22 years: analysis based on uncorrected BMI

| Outcome  at age 22 y | Sex | Unadjusted models | | |  | Sex-adjusted models | | |  | Fully adjusted models^\|\|^ | | |
| --- | --- | --- | --- | --- | --- | --- | --- | --- | --- | --- | --- | --- |
|  |  | ß | 95% CI | *P*-value |  | ß | 95% CI | *P-*value |  | ß | 95% CI | *P*-value |
| BMI^†^ | All^‡^ | 2.09 | 0.68, 3.49 | 0.004 |  | 2.08 | 0.68, 3.49 | 0.004 |  | 1.26 | -0.22, 2.73 | 0.095 |
|  | Male^§^ | 0.66 | -1.62, 2.94 | 0.569 |  |  |  |  |  | 0.54 | -1.88, 2.96 | 0.662 |
|  | Female^§^ | 2.88 | 1.09, 4.67 | 0.002 |  |  |  |  |  | 1.73 | -0.13, 3.59 | 0.068 |
|  |  | OR | 95% CI | *P*-value |  | OR | 95% CI | *P*-value |  | OR | 95% CI | *P*-value |
| Obesity  (BMI ≥30) | All^‡^ | 2.64 | 1.46, 4.76 | 0.001 |  | 2.63 | 1.46, 4.75 | 0.001 |  | 1.88 | 1.00, 3.56 | 0.051 |
|  | Male^§^ | 1.06 | 0.30, 3.01 | 0.917 |  |  |  |  |  | 0.89 | 0.23, 2.79 | 0.850 |
|  | Female^§^ | 4.20 | 2.02, 8.45 | <0.001 |  |  |  |  |  | 2.80 | 1.26, 5.99 | 0.009 |

BMI, Body mass index

^*^ Based on two group-model trajectories (reference category: low-risk of household food insecurity).

^†^ BMI was calculated as kg/m^2^ based on self-reported height and weight.

^‡^  *n* 696; high-risk trajectory, *n* 69. Analyses are based on linear regressions for BMI and on logistical regressions for obesity.

^§^ Male *n* 242; female *n* 454

^||^ Analyses adjusted for maternal education and immigrant status, household income and type of family when QLSCD participants were children (*n* 689).
